# Evolutionary origin and distribution of amino acid mutations associated with resistance to sodium channel modulators in onion thrips, *Thrips tabaci*

**DOI:** 10.1038/s41598-024-54443-9

**Published:** 2024-02-15

**Authors:** Akiya Jouraku, Yui Tomizawa, Kazuki Watanabe, Kiyoshi Yamada, Seigo Kuwazaki, Misato Aizawa, Satoshi Toda, Shoji Sonoda

**Affiliations:** 1 Institute of Agrobiological Sciences, National Agriculture and Food Research Organization, Tsukuba, Ibaraki 305-8634 Japan; 2 School of Agriculture, Utsunomiya University, Utsunomiya, Tochigi 321-8505 Japan; 3 Seisan Regional Agricultural Extension Center, Mitoyo, Kagawa 769-1503 Japan; 4 Institute for Plant Protection, National Agriculture and Food Research Organization, Higashihiroshima, Hiroshima 739-2494 Japan

**Keywords:** Ecology, Evolution

## Abstract

In onion thrips *Thrips tabaci*, reduced sensitivity of the sodium channel caused by several sodium channel mutations have been correlated with pyrethroid resistance. For this study, using mitochondrial cytochrome *c* oxidase subunit I gene sequences, we examined the phylogenetic relation among a total of 52 thelytokous and arrhenotokous strains with different genotypes of the sodium channel mutations. Then, we used flow cytometry to estimate their ploidy. Results showed that the strains are divisible into three groups: diploid thelytoky, triploid thelytoky, and diploid arrhenotoky. Using 23 whole genome resequencing data obtained from 20 strains out of 52, we examined their genetic relation further using principal component analysis, admixture analysis, and a fixation index. Results showed that diploid and triploid thelytokous groups are further classifiable into two based on the sodium channel mutations harbored by the respective group members (strains). The greatest genetic divergence was observed between thelytokous and arrhenotokous groups with a pair of T929I and K1774N. Nevertheless, they shared a genomic region with virtually no polymorphism around the sodium channel gene loci, suggesting a hard selective sweep. Based on these findings, we discuss the evolutionary origin and distribution of the sodium channel mutations in *T. tabaci*.

## Introduction

Although thought to have originated in the eastern Mediterranean region, onion thrips, *Thrips tabaci* (Thysanoptera: Thripidae) is now distributed throughout the world^1^. In fact, *T. tabaci* damages widely diverse ornamental and vegetable crops, particularly plants belonging to *Allium*^1,2^. Adults and larvae of *T. tabaci* suck cell fluids from leaves, stems, flowers, and the surfaces of fruits, thereby causing silvery scars and leaf chlorosis. In addition to that direct damage, *T. tabaci* causes large economic losses by transmitting viruses such as *Tomato spotted wilt virus*^3^ and *Iris yellow spot virus*^4^ in a persistent manner.

Three reproductive modes have been reported for *T. tabaci*: thelytoky, arrhenotoky, and deuterotoky^5,6^. Thelytoky is a type of parthenogenesis by which only females develop from unfertilized eggs^5^. Arrhenotoky is a form of parthenogenesis by which unfertilized eggs develop into haploid males^5^. Deuterotoky, by which females and males are produced from unfertilized eggs, has been reported in the United States^6^. Nevertheless, that parthenogenesis is uncommon and might not be a fixed reproductive mode^7^. In *T. tabaci*, the existence of populations containing diploid and tetraploid individuals has also been reported^8,9^.

Pyrethroids are synthetic insecticides that are structurally derived from naturally occurring pyrethrins which are present in the pyrethrum extract of Chrysanthemum species^10^. Pyrethroids, along with DDT, are classified as sodium channel modulators (Group 3) based on their mode of action (IRAC, https://irac-online.org/). Pyrethroids interact with the sodium channels and modify their normal function by inhibiting channel deactivation and stabilizing the open configuration, resulting in repetitive discharges that quickly engender nervous exhaustion, paralysis, and death^11^. The pyrethroid resistance in *T. tabaci* has been correlated mainly with nerve insensitivity conferred by amino acid mutations (M918T, M918L, T929I, V1010A, L1014F, and K1774N) in the sodium channel^12–16^. A pair of M918T and L1014F conferring a high level of resistance to pyrethroids was encoded heterozygously in thelytokous insects^12^. Both arrhenotokous and thelytokous insects encoded T929I homozygously^12,14–16^. Reportedly, K1774N might enhance pyrethroid resistance in combination with T929I^16^. M918L is reportedly involved in pyrethroid resistance alone or in combination with V1010A^13,16,17^. In both cases, M918L was encoded heterozygously in thelytokous insects. Cytochrome P450 and/or nonspecific esterases also have been reported as additional factors contributing to the pyrethroid resistance of *T. tabaci*^14^.

Previously, we have investigated the relation between the reproductive mode and T929I in *T. tabaci* strains collected from various sites in Japan^14,15^. Results revealed that all arrhenotokous strains encoded the resistant amino acid (isoleucine). However, only a few thelytokous strains encoded isoleucine, possibly because of the higher fitness cost for the reproductive mode. It is particularly interesting that heterozygous insects were extremely rare in sites where insects of both reproductive modes were sympatry inhabited^15^ (Sonoda, unpublished data), suggesting reproductive isolation between the respective reproductive modes. Actually, genetic exchange between the two reproductive modes has been reported, albeit with low frequency^18^. Consequently, it remains unclear whether thelytoky evolved T929I independently or obtained the mutation through gene flow from arrhenotoky. The evolutionary origin of the sodium channel mutations other than T929I reported to date in Japan (M918L, a pair of M918T and L1014F) also remains unclear.

For this study, to elucidate the evolutionary origin and distribution of the sodium channel mutations in *T. tabaci*, we examined the phylogenetic relation and ploidy of thelytokous and arrhenotokous strains with different genotypes for the sodium channel mutation sites. Furthermore, using whole genome resequencing (WGR) data obtained from the strains, we examined their genetic relation. We also examined a genomic region with extremely low genetic diversity, a footprint of a hard selective sweep, in the pyrethroid-resistant strain groups with T929I and K1774N.

## Results

### Reproductive mode determination

The reproductive modes of a total of 52 strains used for this study are presented in Table 1. It is noteworthy that KOC2 and KOC2-3, KOC2442 and KOC2442-2, and TKO-DFR and TKO-SPRR were the same strain but were analyzed in different years (Tables 1, S1). Based on their progeny production, 31 were judged as thelytokous. The remaining 21 were regarded as arrhenotokous. No deuterotokous strain was found in this study.

**Table 1:** *Thrips tabaci* strains used in this study.

Strain	Reproductive mode	Ploidy	Genotype				Collected site	Collected year	Host plant
			T929I + K1774N	M918L	M918T + L1014F	V1010A			
WKY-NTB	TH	3	*SSS*	*SSS*	*SSS*	*SSS*	Kinokawa city, Wakayama pref., Kinki dist. (N34° 26′ E135° 36′)	2022	*Allium cepa*
WKY-K	TH	3	*SSS*	*SSS*	*SSS*	*SSS*	Kinokawa city, Wakayama pref., Kinki dist	2022	*Allium cepa*
MYZ-B	TH	3	*SSS*	*SSS*	*SSS*	*SSS*	Miyazaki city, Miyazaki pref., Kyushu dist. (N31° 90′ E131° 42′)	2022	*Allium cepa*
CHB-M6	TH	3	*SSS*	*SSS*	*SSS*	*SSS*	Ichikawa city, Chiba pref., Kanto dist. (N35° 71′ E139° 92′)	2021	*Allium fistulosum*
HKD38	TH	3	*SSS*	*SSS*	*SSS*	*SSS*	Naganuma town, Hokkaido pref., Hokkaido dist. (N43° 00′ E141° 41′)	2021	*Allium cepa*
TOC-N5	TH	3	*SSS*	*SSS*	*SSS*	*SSS*	Mooka city, Tochigi pref., Kanto dist. (N36° 44′ E140° 01′)	2021	*Allium cepa*
TOC-N1	TH	3	*SSS*	*SSS*	*SSS*	*SSS*	Mooka city, Tochigi pref., Kanto dist	2021	*Allium cepa*
CHB-H6	TH	3	*SSS*	*SSS*	*SSS*	*SSS*	Yokoshibahikari town, Chiba pref., Kanto dist. (N35° 39′ E140° 30′)	2022	*Allium fistulosum*
CHB-OT3	TH	3	*SSS*	*SSS*	*SSS*	*SSS*	Minamiboso city, Chiba pref., Kanto dist. (N35° 02′ E139° 50′)	2021	*Allium cepa*
OSK-I52	TH	3	*SSS*	*SSS*	*SSS*	*SSS*	Izumisano city, Osaka pref., Kinki dist. (N34° 85′ E136° 81′)	2021	*Allium cepa*
CHB-A	TH	3	*SSS*	*SSS*	*SSS*	*SSS*	Yokoshibahikari town, Chiba pref., Kanto dist	2022	*Allium fistulosum*
AKT2-8	TH	3	*SSS*	*SSS*	*SSS*	*SSS*	Happo town, Akita pref., Tohoku dist. (N40° 19′ E140° 02′)	2021	*Allium fistulosum*
KOC50^a,c^	TH	3	*SSS*	*SSS*	*SSS*	*SSS*	Nankoku city, Kochi pref., Shikoku dist. (N33° 57′ E133° 64′)	2011	*Allium fistulosum*
KAG1^a,c^	TH	3	*SSS*	*SSS*	*SSS*	*SSS*	Ayagawa town, Kagawa pref., Shikoku dist. (N34° 24′ E133° 92′)	2015	*Allium cepa*
HKD1^c^	TH	3	*SSS*	*SSR*	*SSS*	*SSS*	Naganuma town, Hokkaido pref., Hokkaido dist	2021	*Allium cepa*
HKD2^c^	TH	nd	*SSS*	*SSR*	*SSS*	*SSS*	Naganuma town, Hokkaido pref., Hokkaido dist	2021	*Allium cepa*
HKD3^c^	TH	3	*SSS*	*SSR*	*SSS*	*SSS*	Naganuma town, Hokkaido pref., Hokkaido dist	2021	*Allium cepa*
ANO^c^	TH	nd	*SSS*	*SSS*	*SSS*	*SSS*	Tsu city, Mie pref., Tokai dist. (N34° 43′ E136° 30′)	2016	*Allium fistulosum*
WKY-NEB	TH	2	*SS*	*SS*	*SS*	*SS*	Kinokawa city, Wakayama pref., Kinki dist	2022	*Phaseolus vulgaris*
KUM-YA	TH	2	*SS*	*SS*	*SS*	*SS*	Yatsusiro city, Kumamoto pref., Kyushu dist. (N32° 50′ E130° 60′)	2022	*Allium fistulosum*
OSK-H2	TH	2	*SS*	*SS*	*SS*	*SS*	Hannan city, Osaka pref., Kinki dist. (N34° 35′ E135° 23′)	2021	*Allium fistulosum*
KAG5-2	TH	2	*RR*	*SS*	*SS*	*SS*	Sanuki city, Kagawa pref., Shikoku dist. (N34° 32′ E134° 17′)	2021	*Asparagus officinalis*
OKY-B	TH	2	*SS*	*SS*	*SS*	*SS*	Okayama city, Okayama pref., Chugoku dist. (N34° 65′ E133° 91′)	2022	*Brassica oleracea var. capitata*
KOC2-2^c^	TH	2	*SS*	*SS*	*SS*	*SS*	Unknown	Unknown	Unknown
KOC2^a,b,c^	TH	2	*RR*	*SS*	*SS*	*SS*	Shimanto town, Kochi pref., Shikoku dist. (N32° 99′ E132° 93′)	2012	*Asparagus officinalis*
KOC2-3^b,c^	TH	nd	*RR*	*SS*	*SS*	*SS*	Shimanto town, Kochi pref., Shikoku dist	2012	*Asparagus officinalis*
HYG-AB	TH	2	*SS*	*SS*	*SS*	*SS*	Awaji city, Hyogo pref., Kinki dist. (N34° 26′ E134° 54′)	2022	*Allium cepa*
HYG-KA	TH	2	*SS*	*SS*	*SS*	*SS*	Kasai city, Hyogo pref., Kinki dist. (N34° 55′ E134° 50′)	2022	*Allium fistulosum*
TOK6^a,c^	TH	nd	*RR*	*SS*	*SS*	*SS*	Mugi town, Tokushima pref., Shikoku dist. (N33° 40′ E134° 25′)	2012	*Allium fistulosum*
KAG5-7^c^	TH	2	*RR*	*SS*	*SS*	*SS*	Sanuki city, Kagawa pref., Shikoku dist	2021	*Asparagus officinalis*
KAG5-12^c^	TH	2	*RR*	*SS*	*SS*	*SS*	Sanuki city, Kagawa pref., Shikoku dist	2021	*Asparagus officinalis*
WKY-M918T^c^	TH	nd	*SS*	*SS*	*SR*	*SS*	Kinokawa city, Wakayama pref., Kinki dist	2007	*Vicia sativa*
MYZ-D	AR	2	*RR*	*SS*	*SS*	*SS*	Miyazaki city, Miyazaki pref., Kyushu dist	2022	*Allium cepa*
WKY-G1	AR	2	*RR*	*SS*	*SS*	*SS*	Gobo city, Wakayama pref., Kinki dist. (N33° 89′ E135° 15′)	2022	*Allium cepa*
NGN-M1	AR	2	*RR*	*SS*	*SS*	*SS*	Matsumoto city, Nagano pref., Chubu dist. (N36° 23′ E137° 97′)	2022	*Allium fistulosum*
KYT-M1	AR	2	*RR*	*SS*	*SS*	*SS*	Kyoto city, Kyoto pref., Kinki dist. (N35° 01′ E135° 76′)	2022	*Allium fistulosum*
KYT-T1	AR	2	*RR*	*SS*	*SS*	*SS*	Kyoto city, Kyoto pref., Kinki dist	2022	*Allium fistulosum*
CHB-TA	AR	2	*RR*	*SS*	*SS*	*SS*	Yokoshibahikari town, Chiba pref., Kanto dist	2022	*Allium cepa*
CHB-TB	AR	2	*RR*	*SS*	*SS*	*SS*	Yokoshibahikari town, Chiba pref., Kanto dist	2022	*Allium cepa*
CHB-H2	AR	2	*RR*	*SS*	*SS*	*SS*	Yokoshibahikari town, Chiba pref., Kanto dist	2022	*Allium cepa*
YMN1	AR	2	*RR*	*SS*	*SS*	*SS*	Kai city, Yamanashi pref., Chubu dist. (N35° 66′ E138° 51′)	2022	*Allium fistulosum*
YMG1-1	AR	2	*RR*	*SS*	*SS*	*SS*	Sagae city, Yamagata pref., Tohoku dist. (N38° 38′ E140° 27′)	2022	*Allium fistulosum*
YMG2-1	AR	2	*RR*	*SS*	*SS*	*SS*	Shonai town, Yamagata pref., Tohoku dist. (N38° 50′ E139° 54′)	2022	*Allium fistulosum*
AKT1-A3	AR	2	*RR*	*SS*	*SS*	*SS*	Noshiro city, Akita pref., Tohoku dist. (N40° 12′ E140° 01′)	2021	*Allium fistulosum*
KAG3-5	AR	2	*RR*	*SS*	*SS*	*SS*	Kan-onji city, Kagawa pref., Shikoku dist. (N34° 12′ E133° 66′)	2021	*Allium cepa*
KAG4-5^c^	AR	2	*RR*	*SS*	*SS*	*SS*	Mitoyo city, Kagawa pref., Shikoku dist. (N34° 18′ E133° 71′)	2021	*Allium cepa*
KOC2442^a,b,c^	AR	2	*RR*	*SS*	*SS*	*SS*	Nankoku city, Kochi pref., Shikoku dist	2011	*Allium fistulosum*
KOC2442-2^b,c^	AR	nd	*RR*	*SS*	*SS*	*SS*	Nankoku city, Kochi pref., Shikoku dist	2011	*Allium fistulosum*
KOC16^a,c^	AR	2	*RR*	*SS*	*SS*	*SS*	Nankoku city, Kochi pref., Shikoku dist	2011	*Allium fistulosum*
TKO^c^	AR	nd	*RR*	*SS*	*SS*	*SS*	Tsukuba city, Ibaraki pref., Kanto dist. (N36° 08′ E140° 07′)	2015	*Allium fistulosum*
TKO-DFR^b,c^	AR	nd	*RR*	*SS*	*SS*	*SS*	Tsukuba city, Ibaraki pref., Kanto dist	2018	*Allium fistulosum*
TKO-SPRR^b,c^	AR	nd	*RR*	*SS*	*SS*	*SS*	Tsukuba city, Ibaraki pref., Kanto dist	2018	*Allium fistulosum*
KTF-SPSS^c^	AR	2	*RR*	*SS*	*SS*	*SS*	Kyoto city, Kyoto pref., Kinki dist	2019	*Allium fistulosum*
KTF-SPRR^c^	AR	2	*RR*	*SS*	*SS*	*SS*	Kyoto city, Kyoto pref., Kinki dist	2019	*Allium fistulosum*
TOK401^a,c^	AR	2	*RR*	*SS*	*SS*	*SS*	Mugi town, Tokushima pref., Shikoku dist	2011	*Allium fistulosum*

*AR* arrhenotoky, *TH* thelytoky, *nd* not determined.

^a^Information was reported earlier by Aizawa et al. (2016).

^b^For their origins, see text.

^c^Strains used to construct a whole-genome sequencing library.

### Phylogenetic analysis

**Figure 1:**
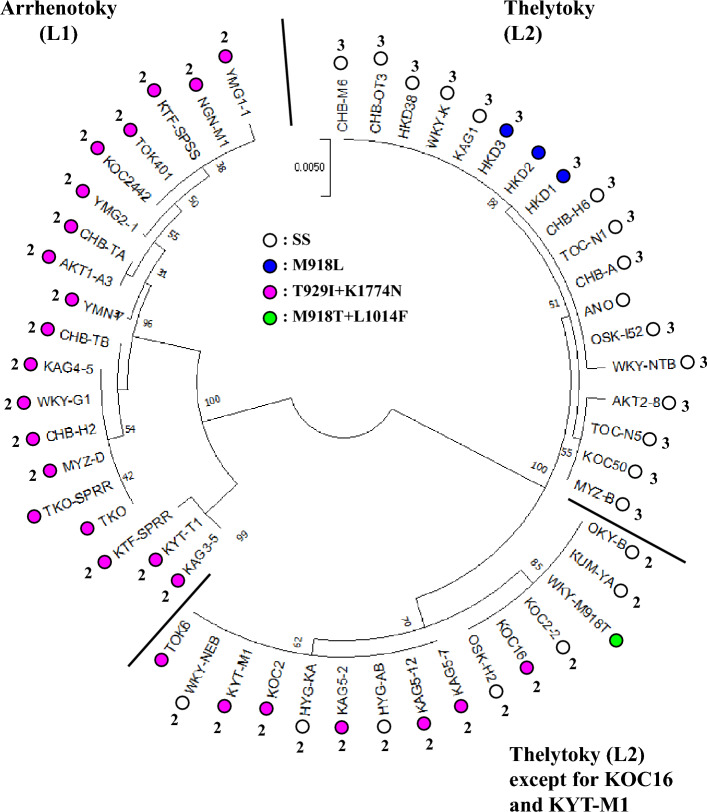
Phylogenetic tree of 52 *Thrips tabaci* strains using the COI gene sequences. The COI gene sequences were obtained through nucleotide sequencing of the PCR products (42 strains other than HKD1, HKD2, HKD3, ANO, TOK6, WKY-M918T, TKO, TKO-SPRR, KTF-SPSS, and KTF-SPRR) and whole genome resequencing data (10 strains other than 42 strains examined using the PCR products). The tree was constructed with the Neighbor-Joining tree method using Molecular Evolutionary Genetics Analysis (MEGA) ver. 10.0. Bootstrap values on nodes were obtained by 1000 replications.

The phylogenetic relation among 52 strains is presented in Fig. 1. The cytochrome *c* oxidase subunit I (COI) sequences were obtained through nucleotide sequencing of the PCR products (GenBank/EMBL/DDBJ accession nos. LC771493-LC771534) and/or WGR (accession nos. LC779518-LC779534). In the latter processes, we found the presence of multiple haplotypes in two thelytokous strains (ANO and HKD2) derived from a single adult female, indicating heteroplasmy as reported^19,20^. Multiple haplotypes were also observed in three arrhenotokous strains (TKO, TKO-SPRR, and KTF-SPRR). However, they might not be heteroplasmic because of their non-isogenic origin (see *Methods*). Among the five strains, TKO-SPRR had four haplotypes, with proportions of 59%, 15%, 14%, and 12% (Table S2). Two haplotypes were found in the remaining four strains. The proportions of the predominant haplotypes for ANO, HKD2, TKO, and KTF-SPRR were, respectively, 57%, 83%, 87%, and 85% (Table S2). Strains of thelytoky, the only reproductive mode identified in India, included not only thelytokous-associated haplotypes but also arrhenotokous-associated haplotypes with low frequencies^20^. The nucleotide sequences of the minor haplotypes of the five strains exhibiting heteroplasmy examined for this study reflected the reproductive modes from which they derived. Nevertheless, in this study, minor sequences detected using WGR were excluded from the phylogenetic analysis. Results demonstrated that 52 strains were divisible into three groups. This finding was strongly supported by the high bootstrap value which we found: 100%. The first and second groups respectively comprise 19 arrhenotokous and 18 thelytokous strains. The third group included 13 thelytokous strains and two arrhenotokous strains (KOC16 and KYT-M1).

### Ploidy determination using flow cytometry

**Figure 2:**
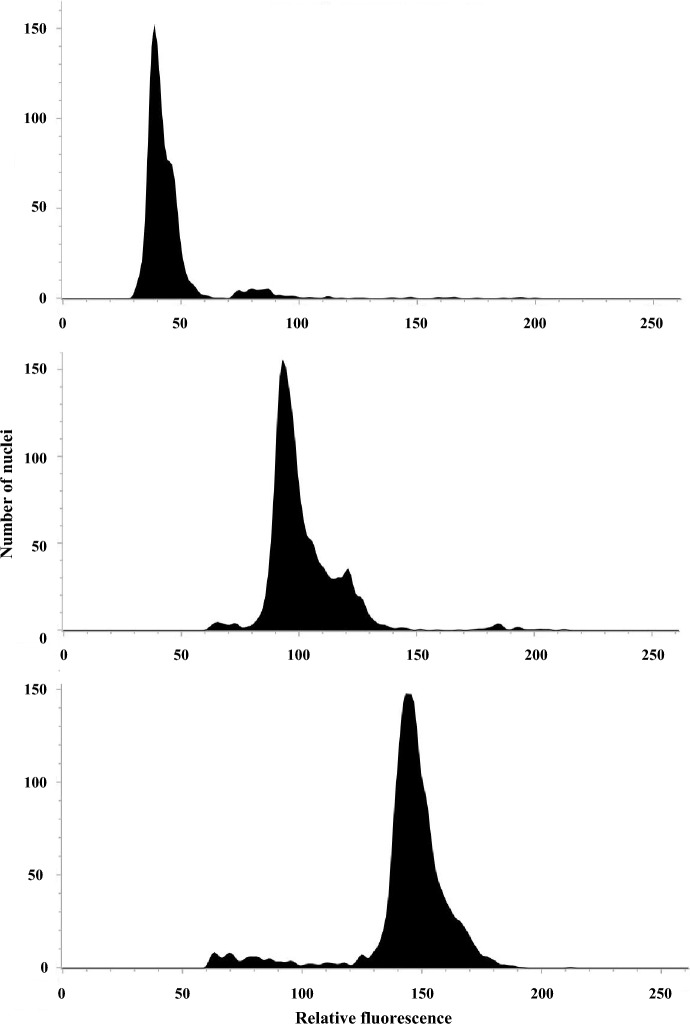
Flow cytometric histogram showing the relative fluorescence of nuclei prepared from haploid males, diploid females, and triploid females of *Thrips tabaci*. Ploidy determination using flow cytometry was conducted for 46 strains, as shown in Table 1.

The ploidy of 46 strains is presented in Fig. 2 (data for CHB-H2, YMG1-1, and WKY-NTB obtained using flow cytometry are shown as examples) and Table 1. Females of all 19 examined arrhenotokous strains and those of 11 examined thelytokous strains were estimated as diploid. Females of the remaining 16 thelytokous strains were estimated as triploid. No arrhenotokous strain estimated as triploid was observed in this study. In the United States, in addition to diploids, the presence of tetraploids that were speculated to have undergone a genome reduction was reported not only in thelytokous insects but also in arrhenotokous insects^8,9^. To clarify the presence or absence of genome reduction, cytological observations of chromosomes must be conducted for thelytokous strains estimated as triploid in this study. Nevertheless, in this study, these thelytokous strains with larger genome sizes are treated as triploid because of the lack of contradictory observations.

### Genotyping of sodium channel mutations

Genotypes of the amino acid mutation sites in the sodium channel (M918T, M918L, T929I, L1014F, V1010A, and K1774N) of 52 strains are presented in Table 1. Only one strain collected in 2007 (WKY-M918T) encoded M918T and L1014F heterozygously (data for the M918T site depicted in Fig. S1). All 21 arrhenotokous strains (Both KOC2442 and KOC2442-2 and TKO-DFR and TKO-SPRR were counted as one strain.) were resistant homozygotes for the T929I and K1774N sites. Five thelytokous strains (KAG5-2, KAG-5–7, KAG5-12, KOC2, and TOK6) were also resistant homozygotes for both sites. HKD1, HKD2, and HKD3 encoded M918L heterozygously (data for HKD1 depicted in Fig. S1). No insect encoding V1010A was observed.

### WGR and SNP calling

Sufficient whole-genome resequencing data were generated and mapped to the reference genome sequences for each strain (depth of coverage and mapped ratio were 176.1 and 83.0% in average respectively (Table S1). The high-quality 1,910,949 biallelic SNPs were called among the 20 strains (23 analyses) selected to include different reproductive modes, ploidy, and sodium channel mutations. The LD-pruned 185,335 biallelic SNPs were extracted.

### Principal component analysis (PCA) and admixture analysis

**Figure 3:**
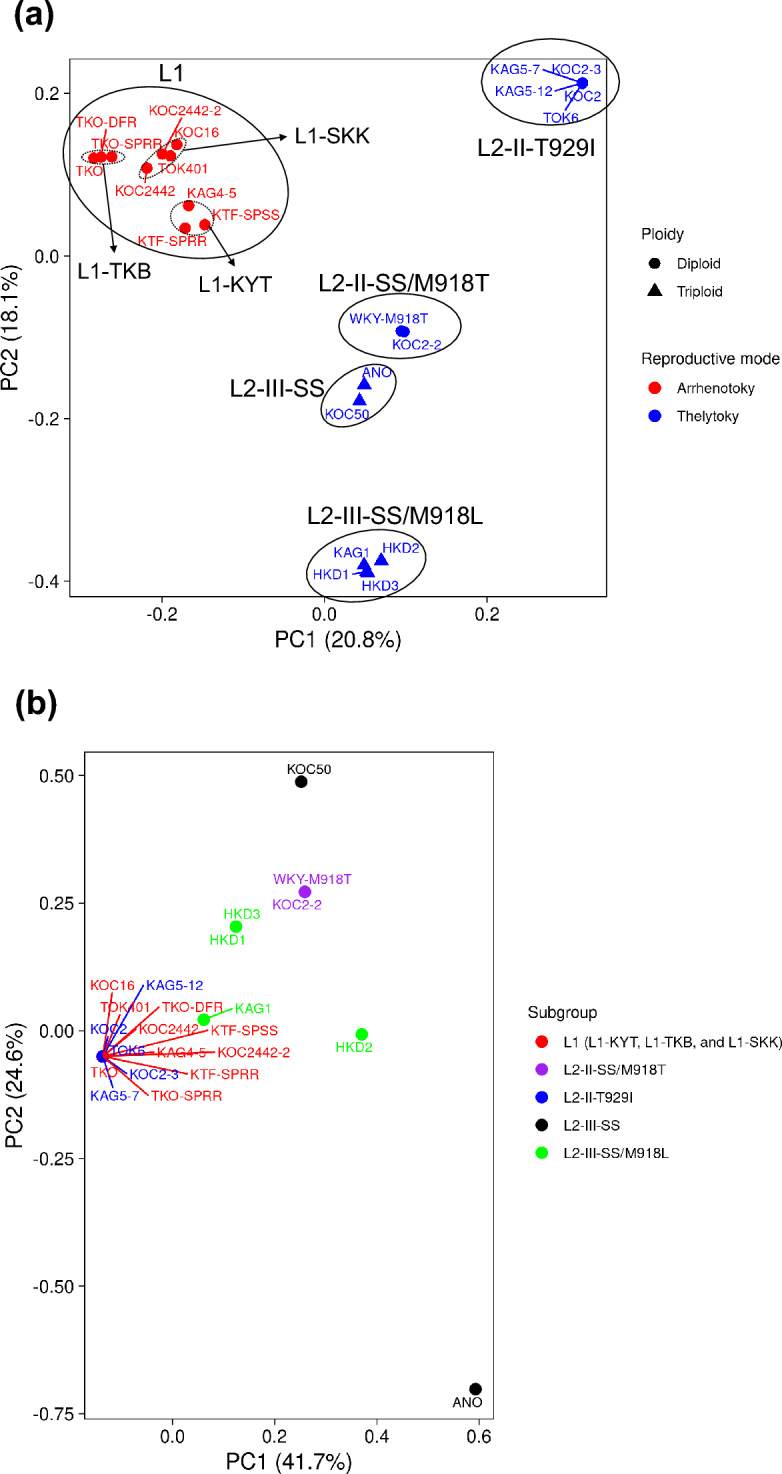
PCA plot of 20 strains (23 analyses) of *Thrips tabaci* using biallelic linkage disequilibrium (LD) pruned SNP data: (**a**) PCA plot based on 185,335 SNPs in the whole genome; (**b**) PCA plot based on 36 SNPs in the voltage-gated sodium channel gene locus (TTG013117).

PCA analysis based on SNP of genome resequencing was conducted using 12 thelytokous strains (13 analyses) and eight arrhenotokous strains (10 analyses) (Fig. 3a and Table S3). The ploidy of TKO-SPRR, TKO, TOK6, WKY-M918T, ANO, and HKD2 is unknown. However, in this analysis, the first four were treated as diploid and the last two as triploid based on the results of phylogenetic analysis (Fig. 1). Results revealed clear segregation between reproductive modes. Results also revealed that thelytokous strains were segregated into four subgroups: diploids with T929I and K1774N (hereinafter designated as L2-II-T929I, four strains (five analyses)), triploids with no mutations (hereinafter, L2-III-SS, two strains), triploids with M918L (hereinafter, L2-III-SS/M918L, four strains) in which one strain (KAG1) has no M918L mutation, and others (hereinafter, L2-II-SS/M918T, two strains) in which one strain had a pair of M918T and L1014F. Segregation was also observed among arrhenotokous strains: L1-TKB (two strains (three analyses)), L1-KYT (three strains), and L1-SKK (three strains (four analyses)). The arrhenotokous strains were sub-grouped based on the collected area, except for KAG4-5. KAG4-5 was derived from the area from which L1-SKK originated, but was classified into L1-KYT. PCA analysis of sodium channel gene sequences from the 20 strains (23 analyses) described above showed virtually no polymorphism among strains with T929I and K1774N (strains in the L1-TKB, L1-KYT, L1-SKK, and L2-II-T929I subgroups) (Fig. 3b and Table S4).

**Figure 4:**
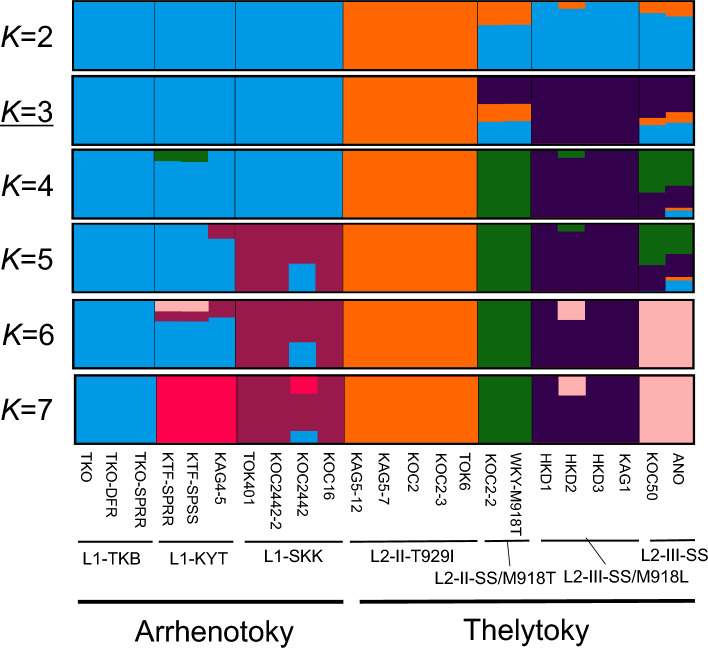
Estimated ancestry proportion of 20 strains (23 analyses) of *Thrips tabaci* by admixture analysis. LD-pruned 185,335 SNPs were used. Results from *K* = 2 to 7 (the number of ancestral populations) were evaluated. *K* = 3 with the lowest CV error value (Fig. S2) was regarded as the best *K* value. Each column represents one strain (corresponding to one pooled whole genome resequencing data). Each *T. tabaci* subgroup (L1-TKB, L1-KYT, L1-SKK, L2-II-T929I, L2-II-SS/M918T, L2-III-SS/M918L, and L2-III-SS) is separated by each vertical bar.

The genetic structure of thelytokous and arrhenotokous strains by admixture analysis is presented in Fig. 4. The CV error estimate results revealed an optimum value of *K* as three (CV error = 0.290). Similar low CV error (0.300) was observed at *K* of four (Fig. S2). In fact, no admixture was observed among the strains belonging to the L1 (L1-TKB, L1-KYT, and L1-SKK), L2-II-T929I, and L2-III-SS/M918L subgroups at *K* = 3. By contrast, the L2-II-SS/M918T and L2-III-SS subgroups showed similar admixture with the L1 (ancestry proportion of 0.283–0.336), L2-II-T929I (0.102–0.267), and L2-III-SS/M918L (0.405–0.614) subgroups. Furthermore, admixture with the L2-II-SS/M918T subgroup was slightly observed (ancestry proportion is 0.154–0.167) in two strains in the L1-KYT subgroup (KTF-SPSS and KTF-SPRR) and was highly observed (0.526–0.627) in the L2-III-SS subgroup at *K* = 4. The estimated ancestry classification at *K* = 7 almost corresponds to the seven subgroups based on the PCA analysis.

### Population differentiation analysis

The results of fixation index (*F*_ST_) analysis are presented in Table 2. A consistently high degree of genetic divergence was observed among the three L1 subgroups and the L2-II-T929I subgroup (*F*_ST_ = 0.3389–0.4461). The L2-II-T929I subgroup was also highly diverged from the other L2 subgroups (L2-II-SS/M918T, L2-III-M918L, and L2-III-SS) (*F*st = 0.3356–0.4171). The L1-KYT subgroup showed smaller genetic divergence with the two L2 subgroups (L2-III-SS and L2-II-SS/M918T) (*F*_ST_ = 0.1043 and 0.1698) than the other L1 subgroups (*F*_ST_ = 0.2032–0.3007). Genetic divergence between the L1-KYT and the other two L1 subgroups was less (*F*_ST_ = 0.0890 and 0.0912) than that found between the L1-TKB and L1-SKK subgroups (*F*_ST_ = 0.1657). In fact, it was almost equal to that between the L1-KYT and L2-II-SS/M918T subgroups (*F*_ST_ = 0.1698). Among the L2 subgroups other than the L2-II-T929I subgroup, genetic divergence between the L2-III-SS and L2-II-SS/M918T subgroups was smaller (*F*_ST_ = 0.0825) than any of the other combinations (*F*_ST_ = 0.1817 and 0.2861).

**Table 2:** Pairsise comparision of the fixation index (*F*_ST_) values of three arrhenotokous subgroups and four thelytokous subgroups using genome-wide 1,910,949 biallelic SNPs.

Subgroup	L1-KYT	L1-TKB	L1-SKK	L2-II-SS/M918T	L2-III-SS/M918L	L2-III-SS
L2-II-T929I	***0.3389***	***0.4461***	***0.3932***	***0.3902***	***0.4171***	***0.3356***
L1-KYT	–	*0.0912*	*0.0890*	*0.1698*	**0.2194**	*0.1043*
L1-TKB		–	*0.1657*	***0.3007***	***0.3067***	**0.2032**
L1-SKK			–	**0.2726**	**0.2856**	**0.2137**
L2-II-SS/M918T				–	**0.2861**	*0.0825*
L2-III-SS/M918L					–	*0.1817*

For strains in each subgroup, see text.

For convenience, the backgrounds of the *F*_ST_ values below 0.2, between 0.2 and 0.3, and above 0.3 are italic, bold and bold italic, respectively.

### Positive selection scan

**Figure 5:**
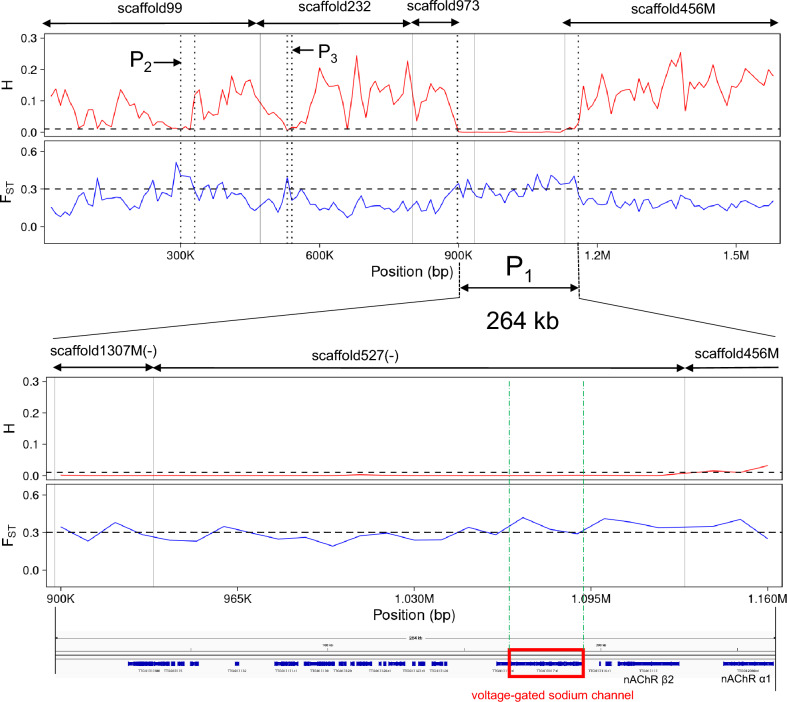
Selective-sweep region including the sodium channel gene. Distribution of the expected heterozygosity (*H*) and fixation index (*F*_ST_) for non-overlapping 10 kb sliding windows in 1,588,560 bp genomic region with estimated conserved synteny to the part of chromosome 15 in *Thrips palmi* (upper row) and in the 264 kb hard selective sweep region (lower row). Three regions surrounded with dotted black vertical bars represent selective sweep regions (P_1_, P_2_, and P_3_). Thin solid black bars represent border lines of scaffolds. One region surrounded with a green dotted vertical bar represents a genomic region of the sodium channel gene. Overlapped regions with scaffold572 were removed manually in scaffold1303M and scaffold456M. The three scaffolds were concatenated manually (scaffold1303M and scaffold527 were aligned to minus strand). The order of scaffold 99, scaffold232, scaffold973, and the concatenated three scaffolds was estimated based on the conserved synteny among the predicted genes in these scaffolds and genes in the chromosome 15 of *Thrips palmi* (Table S7).

Eight candidate regions of selective sweep were detected in eight scaffolds (Table 3). Among them, regions with extremely low genetic diversity (avg. *H* < 0.001) were detected in scaffold527 (170 kb) and scaffold1307 (40 kb). Smaller candidate sweep regions (10–30 kb with avg. *H* = 0.023–0.0107) were detected in the other six scaffolds. We compared the 5′/3′-end nucleotide sequences of the eight scaffolds with those of whole scaffolds using blastn search (e-value < 1e-100) and identified overlapped regions among scaffold1307, scaffold527 and scaffold456, resulting in one concatenated region with these scaffolds (Fig. 5). Furthermore, we compared the sequences of predicted genes in the eight scaffolds with genes in the closely related *Thrips palmi* (available at https://ftp.ncbi.nlm.nih.gov/genomes/all/GCF/012/932/325/GCF_012932325.1_TpBJ-2018v1/) by blastp search (e-value < 1e-5) and found that genes in six scaffolds other than scaffold284 and scaffold852 in Table 3 share highly conserved synteny with *T. palmi* genes in the chromosome 15 (Fig. S3 and Table S5). Based approximately on the homologous genes in *T. palmi*, we estimated that two scaffolds in Table 3 (scaffold99 and scaffold232) and scaffold973 (no putative sweep region) were likely to be located next to the concatenated region with extremely low genetic diversity, resulting in a 1.58 Mb region with conserved synteny to a part of chromosome 15 in *T. palmi* (Fig. 5). The region is referred to as chr15p later in this report.

**Table 3:** Candidate selective sweep regions detected by positive selection scan.

No	Scaffold	Start pos. (bp)	End pos. (bp)	Region size (kb)	Avg. *H*	Avg. *F*_ST_	Min. *H*	Max.* F*_ST_	No. of genes
1	scaffold527	1	1,70,000	170	0.0002	0.3065	0.0000	0.4142	20
2	scaffold1307	1	40,000	40	0.0003	0.3288	0.0000	0.3789	2
3	scaffold372	3,80,001	3,90,000	10	0.0023	0.3311	0.0023	0.3311	3
4	scaffold284	4,10,001	4,30,000	20	0.0030	0.3085	0.0000	0.3155	3
5	scaffold456	20,001	30,000	10	0.0040	0.3967	0.0040	0.3967	1
6	scaffold232	60,001	70,000	10	0.0048	0.3675	0.0048	0.3675	2
7	scaffold852	1	10,000	10	0.0087	0.3529	0.0087	0.3529	1
8	scaffold99	3,00,001	3,30,000	30	0.0107	0.4025	0.0066	0.4080	2

Consequently, six candidate regions of the selective sweep were detected, shown as P_1_-P_6_ in Fig. 5 and Fig. S4. Among them, the 264 kb region (P_1_) with almost no genetic diversity in *T. tabaci* strains with T929I was the largest and was therefore detected as a signature of a hard selective sweep in chr15p (Fig. 5). A gene encoding sodium channel (gene ID: TTG013117), a target of pyrethroids, is located on P_1_ (Fig. 5 and Table S6). Two small candidate sweep regions, P_2_ (30 kb) and P_3_ (10 kb), were also detected in chr15p (Fig. 5). Two genes were found to be located, respectively, in P_2_ and P_3_ (Table S7). Detoxification-related genes, UDP-glucuronosyltransferase (TTG010749.t1) and carboxylesterase (TTG009011.t1), were located in each of two other small candidate sweep regions: P_4_ (20 kb) and P_5_ (10 kb) (Fig. S4 and Table S7). Only one gene was located in another candidate sweep region: P_6_ (10 kb) (Fig. S4 and Table S7).

### Expression levels of two detoxification-related genes in two small candidate sweep regions

To examine the possible involvement of the two detoxification-related genes (TTG010749.t1 and TTG009011.t1) identified in the two small candidate sweep regions in insecticide resistance, we evaluated the expression levels of both genes in two pyrethroid-resistant arrhenotokous strains (IKM2014 and KOC2442) and five pyrethroid-susceptible thelytokous strains (KOC50, OHBO2014, OHBE2014, OKW2014, and IGD2014) using RNA-seq data obtained in our earlier work^16^. The adult females of the seven strains used for the assay had no insecticidal exposure^16^. Results demonstrated that the resistant strains exhibited differential expression (false discovery rate (FDR) < 0.05) relative to the five susceptible strains in the gene TTG009011.t1 (venom carboxylesterase-6-like). No such differential expression was observed in the gene TTG010749.t1 (UDP-glucuronosyltransferase).

## Discussion

*Thrips tabaci* has been divided into three groups based on their COI sequences: tobacco-associated arrhenotokous (T), leek-associated arrhenotokous (L1), and leek-associated thelytokous (L2)^21^. Earlier phylogenetic studies using the COI sequences revealed thelytoky to have been derived only once from arrhenotoky and to have formed a distinct monophyletic group^6,21–23^. Our phylogenetic analysis revealed that a total of 52 strains examined are divisible into three groups, one L1 and two L2 groups, based on the COI sequences having a length of 655 bp. From our ploidy analyses, all arrhenotokous strains were estimated as diploid. Furthermore, one L2 group was estimated as diploid, and the other as triploid. This (sub)grouping of the L2 group reflecting the ploidy was not reported earlier. No strain belonging to the T group was found in this study.

Reportedly, M918L is encoded heterozygously by pyrethroid-resistant thelytokous strains^16^. Results of the present study demonstrated that thelytokous strains encoding M918L (HKD1, HKD2, and HKD3) are possibly triploid. The peak nucleotide sequence intensity correlated with the resistant amino acid (leucine) on the chromatogram obtained through direct sequencing of the sodium channel gene fragment was half or less of that correlated with the susceptible amino acid (methionine) (Fig. S1). On the other hand, in diploid WKY-M918T, the peak nucleotide sequence intensity correlated with the resistant amino acid (threonine) was not much different from that correlated with the susceptible amino acid (methionine) (Fig. S1). These findings suggest that the resistant amino acid (leucine) is encoded on a single chromosome out of three in the three strains with M918L examined for this study.

The presence of M918L in the sodium channel has been reported in some aphids such as *Aphis gossypii*^24,25^, *Myzus persicae*^26,27^, and *Rhopalosiphum padi*^28,29^ in relation to resistance to pyrethroids. In the three aphid species, M918L was singly involved in the resistance. In *T. tabaci*, M918L was observed singly^16^ or in combination with V1010A^13^. All or most insects encoding M918L in the insect species described above were heterozygotes, suggesting the fitness cost of the homozygous form. Reportedly, adult females of the thelytokous strains with T929I exhibited shorter longevity and produced fewer eggs than those of thelytokous strains without T929I and the arrhenotokous strains with T929I^15^, suggesting the larger fitness cost of T929I in the thelytoky. The thelytokous strains heterozygously encoding a pair of M918T and L1014F showed a high degree of resistance to pyrethroids^12^. However, those strains produced fewer eggs than susceptible strains did^30^ (Toda, unpublished data), suggesting the fitness cost of a pair of M918T and L1014F. To evaluate details related to the fitness cost of M918L more precisely, pyrethroid resistance and biotic performances such as development, fecundity, and longevity must be examined using strains with and without M918L.

Li et al.^18^ reported that gene flow occurred from arrhenotokous males to thelytokous females in the laboratory: i.e. the arrhenotokous male-derived haplotype of histone H3 gene was transferred to the progenies with low frequencies through mating with thelytokous females. However, the ploidy of the mating pairs remained unknown. In general, triploid insects are unable to produce normal gametes because of abnormal meiotic synapsis. Their consequent inability to produce normal bivalent chromosomes disrupts the sex determination system. For that reason, gene transfer might have occurred between arrhenotokous males and thelytokous females with diploid ploidy. Considering the results of our admixture analysis, females belonging to the L2-II-SS/M918T subgroup might be the most probable recipients of gene flow from arrhenotokous males. To verify this supposition, mating experiments must be conducted in the future.

In this study, we found almost no polymorphism across 246 kb genomic regions harboring the sodium channel genes in thelytokous and arrhenotokous strains with T929I and K1774N, suggesting a hard selective sweep. However, such selective sweep was not observed in the strains with M918L, as were the cases in the other strains without T929I and K1774N. Heterologous expression of sodium channel with T929I using the *Xenopus* oocyte showed the contribution of the mutation to a high degree of resistance against pyrethroids and DDT^31,32^. By contrast, *M. persicae* carrying M918L revealed resistance to pyrethroids but not to DDT^27^. Mutation from methionine to threonine at the same site (M918T) was shown to involve an extremely high level of resistance against pyrethroids^33^ but not against DDT^34^. These results suggest that the selective sweep observed in the strains with T929I and K1774N is associated with DDT rather than pyrethroids. Similar selective sweeps associated with DDT have been reported in some insects^35^. Calla et al.^35^ found a selective sweep across ca. 500 kb genomic region harboring genes encoding the sodium channel and cytochrome P450 among *Amyelois transitella* populations and its association with exposure to DDT. A selective sweep of ca. 80 genes including the sodium channel gene was also reported in urban populations of *Anopheles gambiae* that were possibly exposed more frequently to xenobiotics including DDT^36^.

From this study, no gene encoding a degradation enzyme possibly associated with insecticide resistance was found in the genomic region showing the hard selective sweep (Table S6). However, two detoxification-related genes (UDP-glucuronosyltransferase and carboxylesterase) were found in two other small selective sweep regions (Fig. S4, Table S7). Of these, the expression level of the carboxylesterase gene (TTG009011.t1) was up-regulated significantly in the arrhenotokous strains with T929I compared to the thelytokous strains without T929I mutation (Fig. S5). In our earlier report, the possible involvement of cytochrome P450 and/or nonspecific esterases in pyrethroid resistance of *T. tabaci* strains with T929I was predicted^14^. The association of the up-regulated carboxylesterase gene identified in the small selective sweep region with exposure to DDT and resistance to insecticides including pyrethroids remains to be examined in future studies.

Historically, thelytoky has been the dominant reproductive mode for *T. tabaci* in Japan^37,38^. Arrhenotoky was reported for the first time in Shimane prefecture, western Japan, in 1988^39^. In the late 1990s to early 2000s, *T. tabaci* began to damage persimmons^40^, increased damage to Satsuma mandarin^41,42^, asparagus^43^, and ornamental plants^44^, and showed diminished insecticide susceptibility^40,44,45^. Arrhenotokous insects with T929I and thelytokous insects with a pair of M918T and L1014F, both of which show pyrethroid resistance, were first reported during the period^12^. Based on those observations, it has been speculated that thelytokous and arrhenotokous insects showing pyrethroid resistance had invaded from overseas^22,39^. In the present study, the degree of genetic divergence between the L2-II-T929I subgroup and the L1 subgroups (L1-TKB, L1-KYT, and L1-SKK) was found to be greater than any other subgroup combination. Results obtained through our admixture analysis also showed the greatest divergence between both subgroups. Consequently, we concluded that both subgroups had independently evolved T929I overseas under selection with DDT and had invaded Japan at about the same time. Since then, invading arrhenotokous insects have expanded their distribution because of the low fitness cost of T929I^15,46^. However, invading thelytokous insects with a high fitness cost of T929I could not expand their distribution compared to arrhenotokous insects, at least on *Allium* plants^15^. Recently, the basic control agents for *T. tabaci* have been changed from pyrethroids to spinosyns (Group 5) (IRAC). Under these circumstances, monitoring the future changes in sodium channel mutation frequencies is of great interest and importance. Investigating the evolutionary origin and subsequent spread and decline of sodium channel mutations correlated with pyrethroid resistance is expected to be invaluable for resistance management strategies in more pest species.

## Methods

### Insects

*Thrips tabaci* strains used for this study are presented in Table 1. Insect breeding and reproductive mode determination were described by Aizawa et al.^15^. *Thrips tabaci* strains other than TKO, TKO-DFR, TKO-SPRR, KTF-SPSS, and KTF-SPRR were established from a single adult female. Insects were maintained with germinated fava bean or garlic scale (*Allium sativum* L.) at 23 °C under a long photoperiod (16L:8D).

Among the 52 strains, 20 strains (23 analyses: KOC2, KOC2-3, TOK6, KAG5-7, KAG5-12, WKY-M918T, KOC2-2, HKD1, HKD2, HKD3, KAG1, KOC50, ANO, TKO, TKO-DFR, TKO-SPRR, KTF-SPSS, KTF-SPRR, KAG4-5, TOK401, KOC2442, KOC2442-2, and KOC16) were subjected to the genomic resequencing described below. The remaining 32 strains and KOC2, KAG5-7, KAG5-12, KOC2-2, HKD1, HKD2, KAG1, KOC50, KAG4-5, TOK401, KOC2442, and KOC16 strains were subjected to DNA extraction as described below.

### DNA extraction

Genomic DNA was extracted from a single adult female using MightyPrep reagent for DNA (Takara Bio. Inc., Kusatsu, Japan) (10 μL/ insect) according to the manufacturer’s recommendations. The supernatant (0.5 μL) was used for subsequent PCR amplification.

Pooled genomic DNA used for genome resequencing by next-generation sequencing (NGS) was extracted from approximately 50–100 adult females for each strain (Table S1). The insects were homogenized in 200 µL of TNESU buffer^47^. Then 1 µL of proteinase K solution (Takara Bio. Inc.) was added. After incubation of the homogenate at 55 °C for 2 h, 100 µL of saturated NaCl was added and mixed well. After centrifugation at 14,000 × *g* for 10 min at 4 °C, the supernatant was transferred to a new tube. The DNA was precipitated with 200 µL of isopropanol. The precipitated DNA was resuspended in 50 µL of TE buffer containing RNase A. The resulting DNA solution was cleaned up by isopropanol precipitation and was resuspended in 20 µL of TE buffer.

### Phylogenetic analysis

For phylogenetic analysis, the COI sequences were amplified by PCR using the primers LCO1490 (5′-ggtcaacaaatcataaagatattgg-3′) and HCO2198 (5′-taaacttcagggtgaccaaaaaatca-3′)^48^ using EmeraldAmp MAX PCR Master Mix (Takara Bio. Inc.). The PCR conditions were 1 cycle of 3 min at 94 °C, followed by 40 cycles of 15 s at 94 °C, 30 s at 50 °C, and 1 min at 72 °C, with final extension of 72 °C for 7 min. Amplified DNA fragments (655 bp) were sequenced directly using primers used for PCR amplification, a dye terminator cycle sequencing kit (v3.1; Applied Biosystems, Waltham, USA), and a DNA sequencer (3500 Genetic Analyzer; Applied Biosystems). The COI sequences identified by WGR were also used for the phylogenetic analysis (Supplementary information). Phylogenetic relations were examined using Molecular Evolutionary Genetics Analysis (MEGA) ver. 10^49^. A Neighbor-Joining tree was constructed using the *p*-distance model, with which 1000 bootstrap replications were conducted.

### Ploidy determination using flow cytometry

Frozen adult females (10 individuals) were ground in 500 μL ice-cold Galbraith’s Buffer (pH 7.0) containing 45 mM MgCl_2_, 20 mM MOPS, 30 mM sodium citrate, and 0.1% (vol/vol) Triton X-100 using a Kontes Dounce Tissue Grinder (DWK Life Sciences, Mainz, Germany). The resulting solutions were filtrated using Partec CellTrics 30 µm (Sysmex Corp., Norderstedt, Germany) and were stained with propidium iodide (BioLegend, San Diego, USA) (5 μg/ml) in darkness on ice for 30 min. The suspensions obtained in the final step were analyzed using the BD FACSLyric Flow Cytometer (BD Biosciences, Franklin Lakes, USA). The cell DNA content was measured using the fluorescent intensity of each sample exposed to a laser at 488 nm wavelength. BD TACSuite Software (BD Biosciences) was used to obtain the nuclei peaks. Flow cytometric DNA histograms of haploid males were used as a reference to ascertain the ploidy level of thelytokous and arrhenotokous females.

### Genotyping of sodium channel mutations

To examine the presence of the sodium channel mutations associated with pyrethroid resistance, the DNA fragments containing the mutation sites were amplified using PCR with the primers Tt-Na-5′-3 (5′-tgagtccgaagttctatttt-3′) and Tt-Na-3′-5 (5′-ggtccgagatctgattcgtc-3′) (for M918T, M918L, T929I, V1010A, and L1014F)^14,15^ and with the primers K1774N_f (5′-agtgctgcgtctcgtcaag-3′) and K1774N_r (5′-aggacaggaggaacgtgatg-3′) (for K1774N)^16^. The PCR conditions to examine the M918T, M918L, T929I, V1010A, and L1014F sites were one cycle of 3 min at 94 °C, 40 cycles of 15 s at 94 °C, 30 s at 60 °C, and 1 min at 72 °C, with final extension of 72 °C for 7 min. Amplified DNA fragments were sequenced directly using the primer Tt-Na-direct-seq4 (5′-gcgaacgtttgctttgatcc-3′)^14,15^. For the K1774N site, the PCR conditions were one cycle of 2 min at 98 °C, 35 cycles of 10 s at 98 °C, 30 s at 58 °C, and 10 s at 68 °C, with final extension of 68 °C for 5 min. Amplified DNA fragments were sequenced directly using K1774N_f. Nucleotide sequencing was conducted as described above.

### WGR

The pooled genomic DNA of each strain was used to construct a whole-genome sequencing library using Illumina TruSeq DNA PCR-Free library (Illumina TruSeq Nano DNA was used only for WKY-M918T because of its small amount of genomic DNA). Each library was sequenced by Illumina HiSeq X 151 bp paired-end or by Illumina NovaSeq 6000 151 bp paired-end (Table S1). The library construction and sequencing were performed by Macrogen Japan Corp. (Tokyo, Japan). The raw data have been deposited in the DDBJ Sequence Read Archive (DRA). The accession nos. are shown in Table S1.

### Trimming, mapping, and SNP calling

Optical duplicates in the generated raw reads of each population were removed using clumpify.sh in BBTools ver. 38.94^50^ with options "dedupe optical dist = 12,000" for NovaSeq 6000 data (dist = 2500 was used for HiSeq X data). Adapters and low-quality bases were subsequently trimmed using Trimmomatic ver. 0.39^51^ with options "ILLUMINACLIP:TruSeq3-PE-2.fa:2:40:15 LEADING:10 TRAILING:10 SLIDINGWINDOW:4:20 MINLEN:50". The quality of the qualified reads was checked using FastQC ver. 0.11.9^52^. Then, the qualified reads were mapped to the reference genome sequences of *T. tabaci* constructed by us (Supplementary information) using Bowtie2 ver. 2.4.4^53^ with options "–no-mixed –no-discordant -X 2000 –rg-id ${STRAIN} –rg SM:${STRAIN} –rg PU:Illumina" where "${STRAIN}" was replaced by the corresponding strain name. SNP calling was performed using GATK ver. 4.2.3^54^ with the mapping data (BAM format) generated using Bowtie2. Variants (SNPs/INDELs) of each strain in gVCF format were called by the GATK HaplotypeCaller with "-ERC GVCF" option. All the generated gVCF files were combined using GATK CombineGVCFs and were subsequently indexed with GATK IndexFeatureFile. The merged variants were filtered using GATK VariantFilter with options "–filter-expression "QD < 2.0 || FS > 60.0 || SOR > 3.0 || MQ < 30.0 || MQRankSum < -12.5 || ReadPosRankSum < -8.0" recommended by GATK for hard-filtering. Finally, biallelic SNPs were extracted by GATK SelectVariants with options "–restrict-alleles-to BIALLELIC –select-type-to-include SNP".

### PCA and admixture analyses

To prune the linkage disequilibrium (LD), the biallelic SNPs were filtered using PLINK ver. 1.90b6.24^55^ with options "–geno 0.1 –indep-pairwise 50 5 0.5 –maf 0.05 –hwe 0.000001". The LD-pruned biallelic SNPs were subjected to PCA analysis and admixture analysis. Then, PCA analysis was performed using PLINK ver. 1.90b6.24 with the "-pca" option. Admixture analysis was performed using ADMIXTURE ver. 1.3.0^56^ with option "–cv = 10" (tenfold cross-validation (CV)) for *K* (the number of ancestral populations) values 1–8. The CV error estimate was performed by plotting the CV error value of each *K* value. Thereby, the best (lowest) *K* value was determined. Admixture structures of *K* values 2–7 were visualized using StructureSelector^57^.

### Population differentiation analysis

Pairwise population differentiation analysis between seven arrhenotokous and thelytokous (strain) subgroups (Table S3) was performed by calculating the average values of *F*_ST_ using VCFTools ver. 0.1.16^58^. The 1,910,949 SNPs extracted using the hard-filtering described above were used as input data.

### Positive selection scan

To identify the genomic region under positive selection related to the insecticide resistance conferred by T929I, the expected heterozygosity (*H*)^59^ of strains with T929I (L1-TKB, L1-KYT, L1-SKK, and L2-II-T929I subgroups) and pairwise *F*_ST_ values among strains with (L1-TKB, L1-KYT, L1-SKK, and L2-II-T929I subgroups) and without T929I (L2-II-SS/M918T, L2-III-SS/M918L, and L2-III-SS subgroups) were calculated with non-overlapping 10 kb sliding windows against the 1,910,949 SNPs. Then *H* was calculated using a Perl script developed in-house; *F*_ST_ was calculated using VCFTools ver. 0.1.16. Sliding windows with fewer than 10 SNPs were ignored. *H* was used to identify genomic regions with very low genetic diversity among the strains with T929I. *F*_ST_ was used to identify genomic regions with high genetic differentiation between the strains with and without T929I. Finally, the genomic regions including one or more sliding windows with* H* < 0.01 and *F*_ST_ > 0.3 were extracted as candidate regions of selective sweeps related to the insecticide resistance conferred by T929I.

### Supplementary Information

Supplementary Figures.

Supplementary Tables.

Supplementary Information.

## Data Availability

All the COI sequences generated from this work are deposited into GenBank/EMBL/DDBJ databases under accession nos. LC771493-LC771534 and LC779518-779534. The metadata generated and analysed during this study is available in its Supplementary Information file.
