# Supplementary material for: Evolutionary origin and distribution of amino acid mutations associated with resistance to sodium channel modulators in onion thrips, Thrips tabaci
Source: Sci Rep. 2024 Feb 15;14:3792. doi: 10.1038/s41598-024-54443-9 (PMC10869772; doi:10.1038/s41598-024-54443-9)
Supplement: Supplementary file 1 — Supplementary Figures. [file 41598_2024_54443_MOESM1_ESM.pptx]

## Slide 1
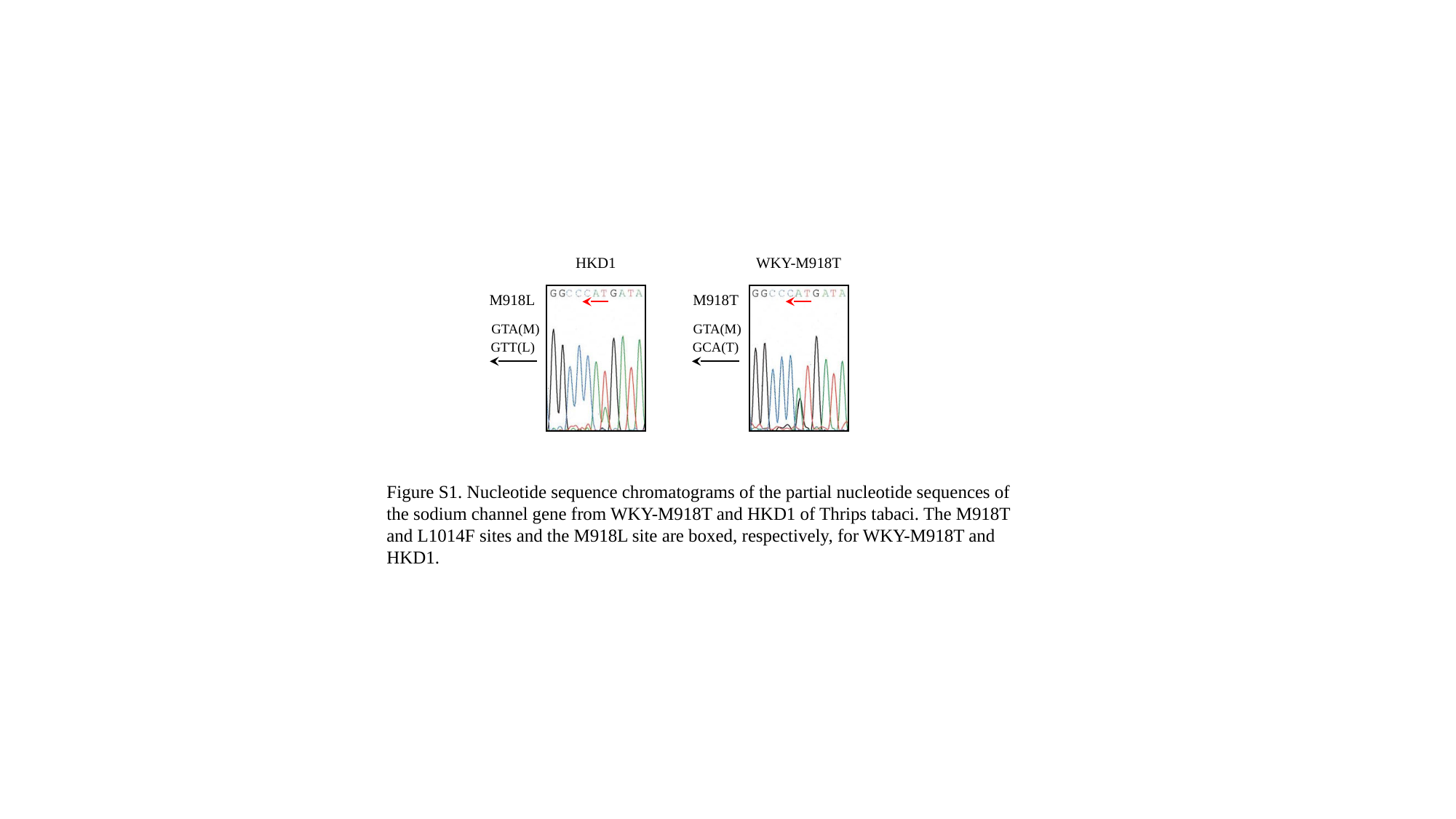

WKY-M918T
HKD1
M918L
M918T
GTA(M)
GTA(M)
GTT(L)
GCA(T)
Figure S1. Nucleotide sequence chromatograms of the partial nucleotide sequences of the sodium channel gene from WKY-M918T and HKD1 of Thrips tabaci. The M918T and L1014F sites and the M918L site are boxed, respectively, for WKY-M918T and HKD1.

## Slide 2
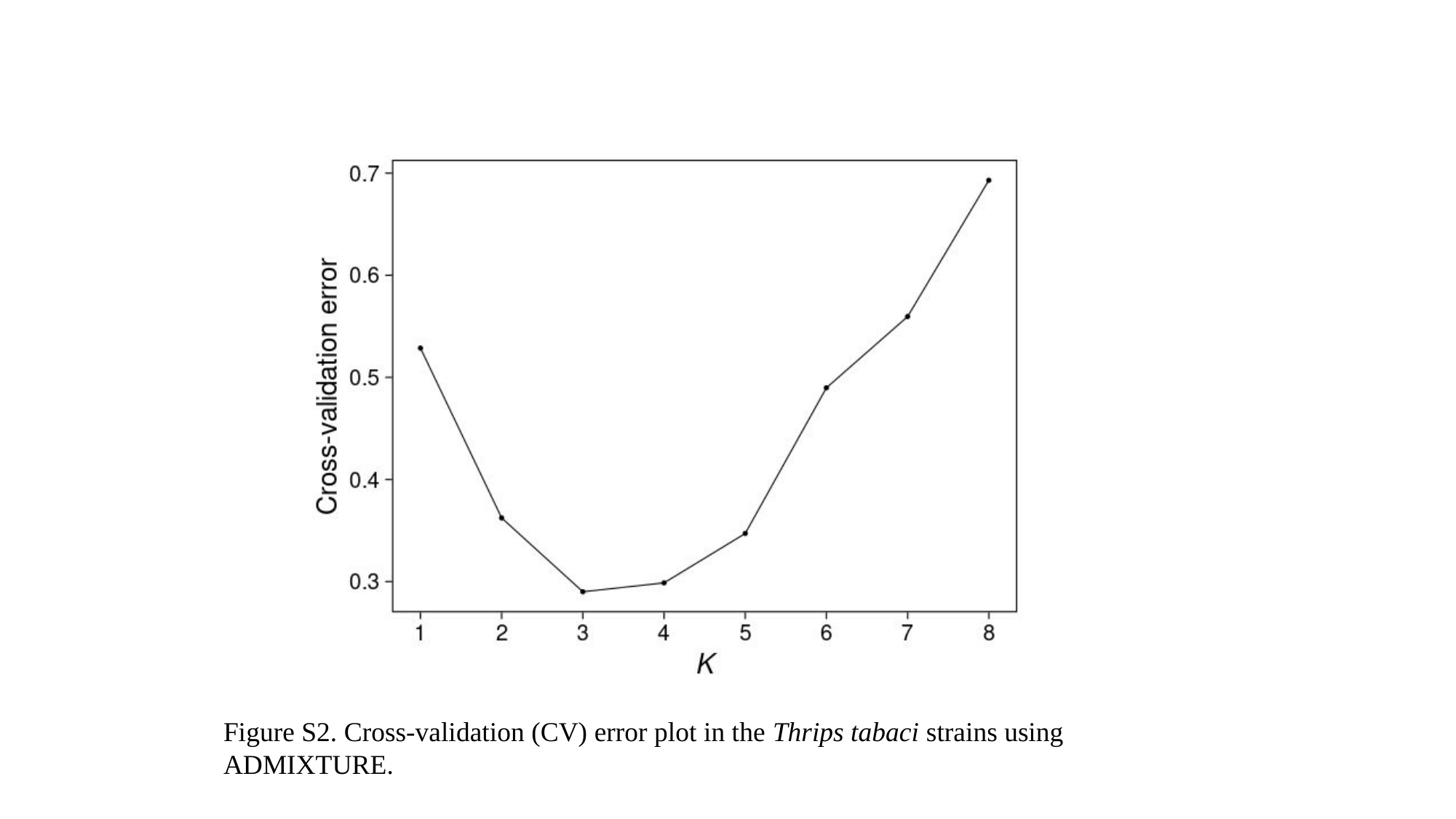

Figure S2. Cross-validation (CV) error plot in the Thrips tabaci strains using ADMIXTURE.

## Slide 3
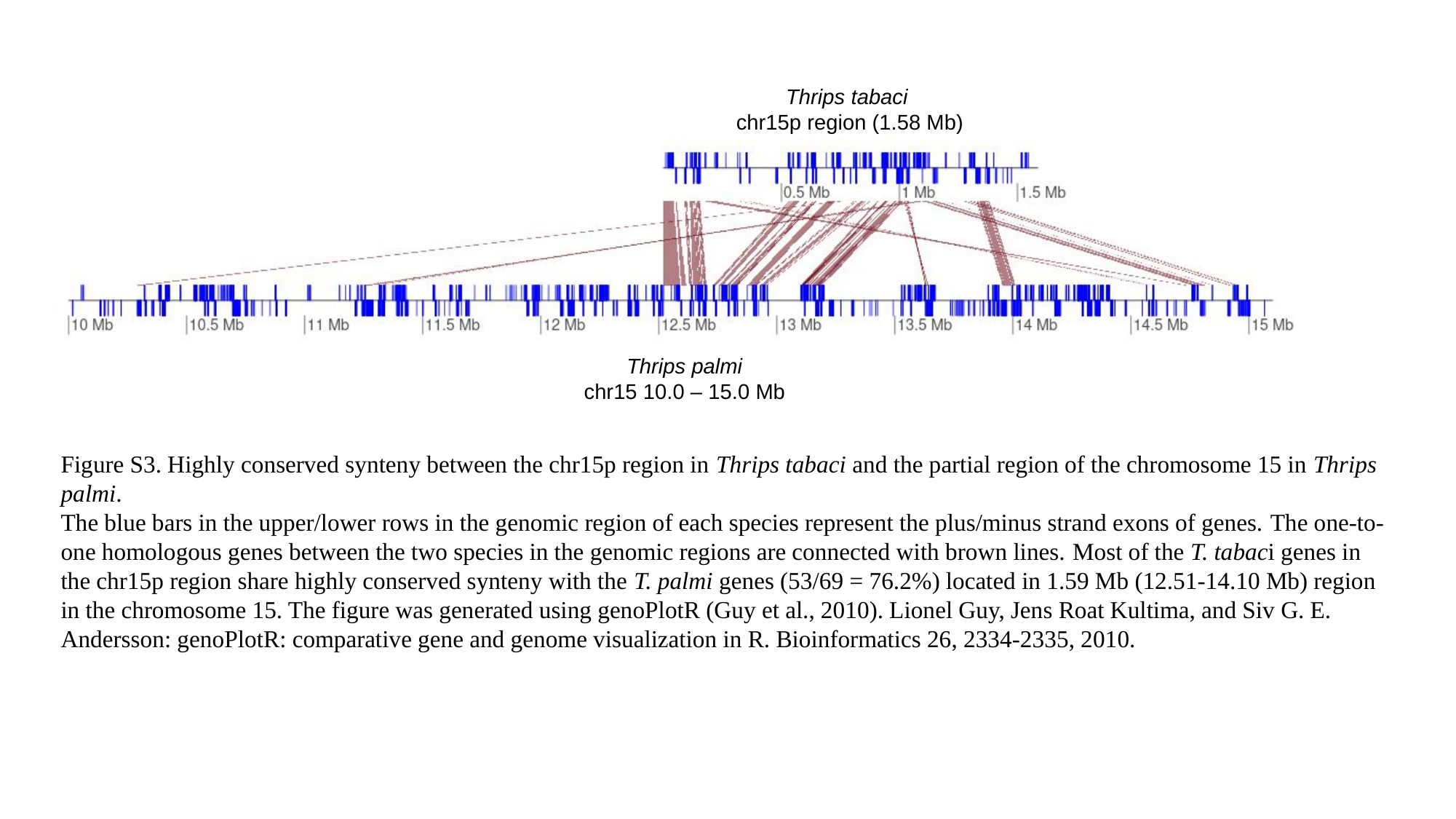

Thrips tabaci
chr15p region (1.58 Mb)
Thrips palmi
chr15 10.0 – 15.0 Mb
Figure S3. Highly conserved synteny between the chr15p region in Thrips tabaci and the partial region of the chromosome 15 in Thrips palmi.
The blue bars in the upper/lower rows in the genomic region of each species represent the plus/minus strand exons of genes. The one-to-one homologous genes between the two species in the genomic regions are connected with brown lines. Most of the T. tabaci genes in the chr15p region share highly conserved synteny with the T. palmi genes (53/69 = 76.2%) located in 1.59 Mb (12.51-14.10 Mb) region in the chromosome 15. The figure was generated using genoPlotR (Guy et al., 2010). Lionel Guy, Jens Roat Kultima, and Siv G. E. Andersson: genoPlotR: comparative gene and genome visualization in R. Bioinformatics 26, 2334-2335, 2010.

## Slide 4
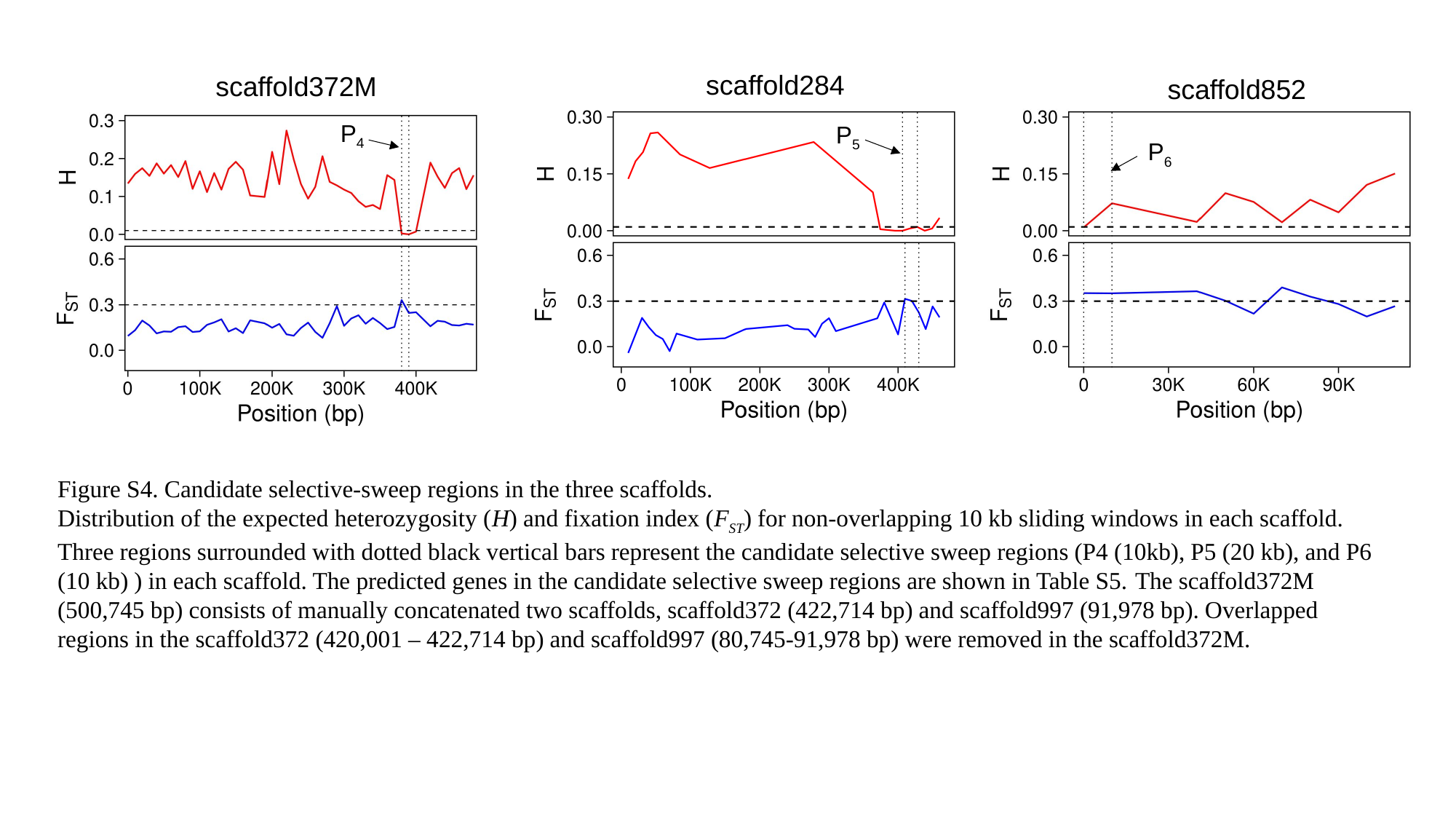

scaffold284
scaffold372M
scaffold852
P4
P5
P6
Figure S4. Candidate selective-sweep regions in the three scaffolds.
Distribution of the expected heterozygosity (H) and fixation index (FST) for non-overlapping 10 kb sliding windows in each scaffold. Three regions surrounded with dotted black vertical bars represent the candidate selective sweep regions (P4 (10kb), P5 (20 kb), and P6 (10 kb) ) in each scaffold. The predicted genes in the candidate selective sweep regions are shown in Table S5. The scaffold372M (500,745 bp) consists of manually concatenated two scaffolds, scaffold372 (422,714 bp) and scaffold997 (91,978 bp). Overlapped regions in the scaffold372 (420,001 – 422,714 bp) and scaffold997 (80,745-91,978 bp) were removed in the scaffold372M.

## Slide 5
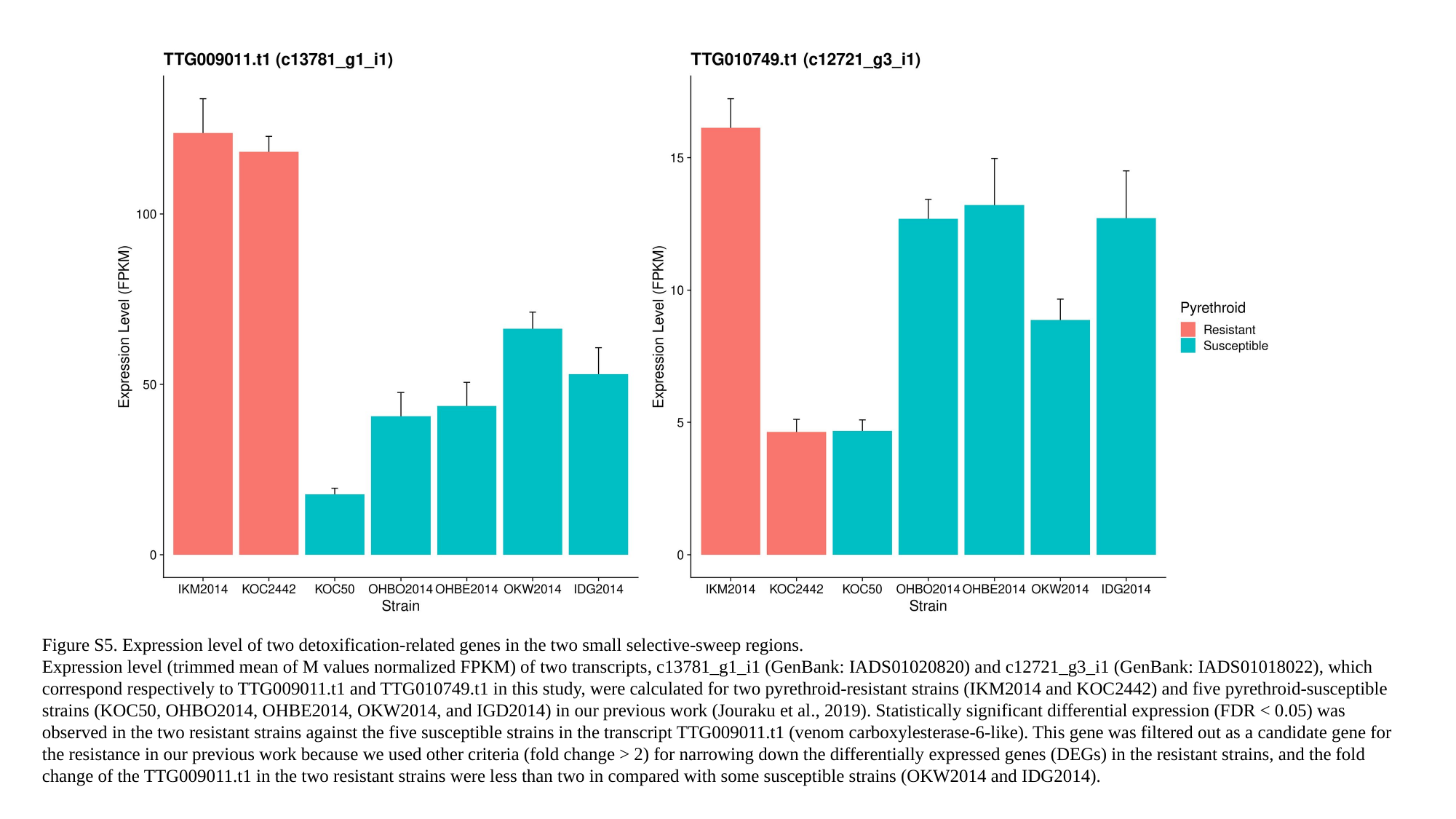

Figure S5. Expression level of two detoxification-related genes in the two small selective-sweep regions.
Expression level (trimmed mean of M values normalized FPKM) of two transcripts, c13781_g1_i1 (GenBank: IADS01020820) and c12721_g3_i1 (GenBank: IADS01018022), which correspond respectively to TTG009011.t1 and TTG010749.t1 in this study, were calculated for two pyrethroid-resistant strains (IKM2014 and KOC2442) and five pyrethroid-susceptible strains (KOC50, OHBO2014, OHBE2014, OKW2014, and IGD2014) in our previous work (Jouraku et al., 2019). Statistically significant differential expression (FDR < 0.05) was observed in the two resistant strains against the five susceptible strains in the transcript TTG009011.t1 (venom carboxylesterase-6-like). This gene was filtered out as a candidate gene for the resistance in our previous work because we used other criteria (fold change > 2) for narrowing down the differentially expressed genes (DEGs) in the resistant strains, and the fold change of the TTG009011.t1 in the two resistant strains were less than two in compared with some susceptible strains (OKW2014 and IDG2014).
