# Supplementary material for: Evolutionary origin and distribution of amino acid mutations associated with resistance to sodium channel modulators in onion thrips, Thrips tabaci
Source: Sci Rep. 2024 Feb 15;14:3792. doi: 10.1038/s41598-024-54443-9 (PMC10869772; doi:10.1038/s41598-024-54443-9)
Supplement: Supplementary file 3 — Supplementary Information. [file 41598_2024_54443_MOESM3_ESM.docx]

**Supplementary Information**

**Evolutional origin and distribution of amino acid mutations associated with resistance to sodium channel modulators in onion thrips, *Thrips tabaci***

**Draft genome assembly of *Thrips tabaci***

A draft genome assembly of *T. tabaci* (accession ID: BTTY01000001-BTTY01001472) used for this study was constructed using long read data from the PacBioRS Sequel system and short read data (paired-end (PE) and mate-pair (MP)) from the Illumina HiSeq system on the ANO strain (thelytokous) (detailed paper is in preparation). Basic statistics of the constructed draft genome assembly are shown in Table S8. Evaluation of the draft genome assembly using BUSCO v5^1^ showed that it covers 98.3% of complete BUSCO genes and 0.7% of fragmented BUSCO genes, whereas only 1.0% of BUSCO genes were missing, indicating good completeness of the draft genome assembly.

Table S8. Basic statistics of the draft genome assembly of *Thrips* *tabaci*

Number of scaffolds 1,472

Total length 329,739,363 bp

N50 348,345 bp

Number of gaps (Ns) 929,969 bp

Average length of scaffolds 224,007 bp

Maximum length of scaffolds 2,632,643 bp

Complete BUSCOs (insecta_db10) 1344 (98.3%)

Complete and single-copy BUSCOs 1282 (93.8%)

Complete and duplicated BUSCOs 62 (4.5%)

Fragmented BUSCOs 10 (0.7%)

Missing BUSCOs 13 (1.0%)

Draft genome assembly evaluation was performed using BUSCO ver. 5.4.4 with the insecta_odb10 dataset (2020-09-10). The numbers of covered BUSCO genes (complete or fragmented) and missing BUSCO genes by the draft genome assembly are shown. Complete BUSCOs are classified into single-copy BUSCOs (only one complete copy was found) and duplicated BUSCOs (two or more complete copies were found).

The genome assembly contains a mitochondrial genome sequence (scaffold1397). The size of the mitochondrial genome sequence is 15,491 bp. The mitochondrial cytochrome c oxidase subunit I (*COI*) gene (1,557 bp) is located at 8136-9692 bp (- strand) in the mitochondrial genome. The haplotypes of mtCOI gene in 14 strains (16 analyses: ANO, HKD1, HKD2, HKD3, M918T, KAG1, KOC50, TOK6, TKO, TKO-DFR, TKO-SPRR, KOC2442, KOC2442-2, KOC16, KTF-SPRR, and KTF-SPSS) were identified using vcf2phylip ver. 2.8^2^ with gVCF format data generated by GATK HaplotypeCaller and CombineGVCFs described in the subsection "2.7 Trimming, mapping, and SNP calling". The frequencies of each haplotype in each strain were calculated manually based on the rate of mapped reads derived from each haplotype.

The predicted gene set of *T. tabaci* was constructed from the draft genome assembly using BRAKER ver. 2.1.6^3,4^ and TSEBRA ver. 1.0.3^5^ using RNA-seq data of four *T. tabaci* strains (TKO, KOC2442, IKM2014, and IDG2014) (accession ID: DRR157291-DRR157293 for KOC2442; DRR157288-DRR157290 for IKM2014; DRR157297-DRR157299 for IDG2014; and DRR508891-DRR508893 for TKO) as hint data. The nucleotide/amino-acid FASTA files and GFF format file of the predicted gene set are available at https://doi.org/10.6084/m9.figshare.24426805. Table S9 presents the basic statistics of the predicted gene set.

Table S9. Basic statistics of the predicted gene set of *Thrips* *tabaci*

Number of genes (protein coding only) 18,965

Number of transcripts 20,384

Total nucleotide numbers 28,269,706 bp

GC content percent 53.26%

N50 1,932 bp

Average length 1,387 bp

Maximum length 40,428 bp

Minimum length 22 bp

Number of genes with blastp (NCBI-nr) hits 16,915

Number of genes with HMMER3 (Pfam DB) hits 13,318

Number of genes with InterProScan5 hits 12,872

ORF sequences of the predicted genes were used to calculate the statistics.

**Supplementary References**

1. Manni, M., Berkeley, M. R., Seppey, M., Simão F. A. & Zdobnov, E. M. BUSCO update: Novel and streamlined workflows along with broader and deeper phylogenetic coverage for scoring of eukaryotic, prokaryotic, and viral genomes. *Mol. Biol. Evol.* **38**, 4647–4654 (2021).

2. Ortiz, E.M. vcf2phylip v2.0: convert a VCF matrix into several matrix formats for phylogenetic analysis (2019). DOI:10.5281/zenodo.2540861

3. Hoff, K. J., Lange, S., Lomsadze, A., Borodovsky, M. & Stanke, M. BRAKER1: Unsupervised RNA-seq-based genome annotation with GeneMark-ET and AUGUSTUS. *Bioinformatics* **32**, 767–769 (2016).

4. Bruna, T., Hoff, K. J., Lomsadze, A., Stanke, M. & Borodovsky, M. BRAKER2: Automatic eukaryotic genome annotation with GeneMark-EP+ and AUGUSTUS supported by a protein database. *NAR Genom. Bioinfor.* **3**, lqaa108 (2021).

5. Gabriel, L., Hoff, K. J., Brůna, T., Borodovsky, M. & Stanke, M. TSEBRA: Transcript selector for BRAKER. *BMC Bioinfor.* **22**, 566 (2021).
